# Analysis of the humoral immune responses among cynomolgus macaque naturally infected with Reston virus during the 1996 outbreak in the Philippines

**DOI:** 10.1186/1746-6148-8-189

**Published:** 2012-10-11

**Authors:** Satoshi Taniguchi, Yusuke Sayama, Noriyo Nagata, Tetsuro Ikegami, Mary E Miranda, Shumpei Watanabe, Itoe Iizuka, Shuetsu Fukushi, Tetsuya Mizutani, Yoshiyuki Ishii, Masayuki Saijo, Hiroomi Akashi, Yasuhiro Yoshikawa, Shigeru Kyuwa, Shigeru Morikawa

**Affiliations:** 1 Special Pathogens Laboratory, Department of Virology 1, National Institute of Infectious Diseases, 4-7-1 Gakuen, Musashimurayama, Tokyo, 208-0011, Japan; 2 Department of Biomedical Science, Graduate School of Agricultural and Life Sciences, University of Tokyo, 1-1-1 Yayoi, Bunkyo-ku, Tokyo, 113-8657, Japan; 3 Department of Virology, Tohoku University Graduate School of Medicine, 2-1 Seiryo-machi, Aoba-ku, Sendai, Japan; 4 Department of Pathology, National Institute of Infectious Diseases, 4-7-1 Gakuen, Musashimurayama, Tokyo, 208-0011, Japan; 5 Department of Microbiology and Immunology, The University of Texas Medical Branch at Galveston, Mary Moody Northen Pavilion 3.206D, 301 University Boulevard Galveston, Texas, TX, 77555-0436, USA; 6 Veterinary Public Health Specialist, Aralia St., Ayala Westgrove Heights, Silang, Cavite, 4118, Philippines; 7 Faculty of Medicine, The University of Tokyo, Bunkyo-ku, Tokyo, Japan; 8 Department of Veterinary Microbiology, Graduate School of Agricultural and Life Sciences, University of Tokyo, 1-1-1 Yayoi, Bunkyo-ku, Tokyo, 113-8657, Japan

**Keywords:** Ebola, Ebolavirus, Reston virus, Reston ebolavirus, Filovirus, Zoonosis, Humoral immune response, Cynomolgus macaque, Cytokine, Antibody

## Abstract

**Background:**

Ebolaviruses induce lethal viral hemorrhagic fevers (VHFs) in humans and non-human primates, with the exceptions of Reston virus (RESTV), which is not pathogenic for humans. In human VHF cases, extensive analyses of the humoral immune responses in survivors and non-survivors have shown that the IgG responses to nucleoprotein (NP) and other viral proteins are associated with asymptomatic and survival outcomes, and that the neutralizing antibody responses targeting ebolaviruses glycoprotein (GP_1,2_) are the major indicator of protective immunity. On the other hand, the immune responses in non-human primates, especially naturally infected ones, have not yet been elucidated in detail, and the significance of the antibody responses against NP and GP_1,2_ in RESTV-infected cynomolgus macaques is still unclear. In this study, we analyzed the humoral immune responses of cynomolgus macaque by using serum specimens obtained from the RESTV epizootic in 1996 in the Philippines to expand our knowledge on the immune responses in naturally RESTV-infected non-human primates.

**Results:**

The antibody responses were analyzed using IgG-ELISA, an indirect immunofluorescent antibody assay (IFA), and a pseudotyped VSV-based neutralizing (NT) assay. Antigen-capture (Ag)-ELISA was also performed to detect viral antigens in the serum specimens. We found that the anti-GP_1,2_ responses, but not the anti-NP responses, closely were correlated with the neutralization responses, as well as the clearance of viremia in the sera of the RESTV-infected cynomolgus macaques. Additionally, by analyzing the cytokine/chemokine concentrations of these serum specimens, we found high concentrations of proinflammatory cytokines/chemokines, such as IFNγ, IL8, IL-12, and MIP1α, in the convalescent phase sera.

**Conclusions:**

These results imply that both the antibody response to GP_1,2_ and the proinflammatory innate responses play significant roles in the recovery from RESTV infection in cynomolgus macaques.

## Background

The family *Filoviridae* includes three genera, Ebolavirus, Marburgvirus, and Cuevavirus. The genus Ebolavirus currently has five members: Bundibugyo virus (BDBV), Ebola virus (EBOV), Reston virus (RESTV), Sudan virus (SUDV), and Taï Forest virus [1]. Filoviruses induce lethal viral hemorrhagic fevers (VHFs) in both humans and non-human primates, while RESTV infection in humans is probably subclinical, yet it also causes highly lethal VHF in macaques [2,3]. RESTV epizootics among cynomolgus macaques emerged in 1989, 1990, 1992, and 1996. In all of these epizootics, the cynomolgus macaques originated in a single primate breeding facility in the Philippines [4]. Although the natural reservoir of RESTV remains unknown, RESTV was isolated from pigs in the Philippines, in addition to porcine reproductive and respiratory syndrome virus (PRRSV) and porcine circovirus type-2 in 2008 [5]. Considering the social impact of ebolaviruses, it is important to understand the endemic and epizootic status of RESTV in the Philippines.

In this study, we investigated the antibody responses of cynomolgus macaques that could be dead-end hosts for RESTV. Using serum specimens collected from cynomolgus macaques during a RESTV outbreak in the Philippines in 1996, we attempted to elucidate the significance of neutralizing antibodies to RESTV in viral clearance. We have previously established an enzyme-linked immunosorbent assay (ELISA) and an indirect immunofluorescent antibody assay (IFA) specific for RESTV nucleoprotein (NP) [6-8]. These assays are useful tools for investigating the signs of RESTV infection in cynomolgus macaques. In human cases, antibody responses against ebolaviruses have been analyzed extensively: IgG responses to NP and other structural proteins (e.g., VP40 and VP35) have been shown to correlate with asymptomatic and surviving cases, and neutralizing antibody responses targeting the ebolaviruses glycoprotein (GP_1,2_) appear to be the major indicator of protective immunity [9].

On the other hand, proinflammatory cytokines/chemokines are known to play a major role in the pathogenesis of ebolaviruses infections in various species. Previous studies have shown an uncontrolled secretion of proinflammatory cytokines/chemokines to contribute to a fatal outcome in EBOV-infected humans [10] and cynomolgus macaques [11]. Strong proinflammatory cytokine/chemokine responses are also observed in convalescent or asymptomatic cases [12,13]. In RESTV-infected cynomolgus macaques, high viremia has been shown to induce the secretion of proinflammatory cytokines/chemokines [14]. However, there have so far only been a limited number of studies on the impact of proinflammatory cytokine/chemokine responses in the convalescent phase of RESTV infection.

In this study, we grouped the cynomolgus macaque samples based on the presence of RESTV NP-antigen in sera and analyzed the antibody reactions and cytokine/chemokine inductions to evaluate the presence of neutralizing antibody to RESTV. We found that the anti-GP_1,2_ responses, but not the anti-NP responses, were closely correlated with the neutralization antibody responses, as well as the clearance of viremia, in the sera of RESTV-infected cynomolgus macaques. Additionally, a high concentration of proinflammatory cytokines/chemokines was detected in the convalescent phase specimens. These data suggest that both the anti-GP_1,2_ responses and proinflammatory cytokines/chemokines play significant roles in the recovery from RESTV infection in cynomolgus macaques.

## Results

### RESTV NP-and GP_1,2_-specific antibodies, neutralizing antibody responses, and the viral antigens in the cynomolgus macaque sera from the 1996 RESTV epizootic

Twenty-seven serum samples derived from cynomolgus macaques that were either found already dead or had been euthanized at the facility were available. The presence of RESTV NP antigens was evaluated by antigen-capture ELISA [15] or immunohistochemistry [3], while that of anti-RESTV NP IgG was evaluated using IgG ELISA and IFA methods [6-8]. RESTV NP antigens were detected in the liver in # 2182, 2612, 2615, 2669, 2739, 2921, 2644 and 2728, while RESTV NP was detected by antigen capture ELISA in the sera of #2182, 2612, 2408, 2615, 2669, 2739, 2921, 2721 and 2972. We therefore assumed that these cynomolgus macaques had suffered from the acute viremic phase of the disease. Seventeen of the 27 samples (#2408, 2615, 2669, 2739, 2921, 2728, 2180, 2181, 2189, 2190, 2191, 2195, 2404, 2693, 2696, 2713 and 2194) were positive for anti-NP IgG in IgG ELISA, while these samples all reacted in IFA. On the other hand, two samples (#2644 and 2719) were only positive in IFA. We considered the samples as anti-NP IgG-positive when either ELISA or IFA showed positive reaction. As a result, a total of 19 samples had anti-NP IgG. Cynomolgus macaques with anti-NP IgG consisted of NP antigen-positive (#2408, 2615, 2669, 2739, 2921, 2644 and 2728) and NP antigen-negative groups (#2180, 2181, 2189, 2190, 2191, 2195, 2404, 2693, 2696 and 2713).

In order to examine whether the sera contained anti-GP_1,2_ antibodies, we employed a GP_1,2_-specific ELISA and IFA [16]. RESTV GP_1,2_ΔTM prepared by a baculovirus expression system and RESTV GP_1,2_-expressing HeLa cells were used as antigens for GP_1,2_-specific ELISA and IFA, respectively. Anti-RESTV GP_1,2_ IgG were detected in 10 (#2180, 2181, 2189, 2190, 2191, 2195, 2404, 2693, 2696 and 2713) out of the 27 serum samples according to ELISA (37%), whereas the remaining 17 samples (63%) showed negative reactions. Nine serum samples positive for GP_1,2_ antibodies in the IgG-ELISA also showed positive reactions in the IFA, while one serum sample (#2194) was only positive in the IFA. Serum samples showing positive reactions in either the GP_1,2_-specific IgG-ELISA or IFA were considered to be anti-GP_1,2_ positive (11/27, 41%, Table 1). Interestingly, the sera derived from cynomolgus macaques in the acute viremic phase did not contain any detectable anti-GP_1,2_ IgG, although they often contained anti-NP IgG.

**Table 1:** Antibody responses and viremic status of Reston virus-infected cynomolgus macaques

**Case ID**	**Anti-NP IgG**		**Anti-GP**_**1,2**_**IgG**		**NT**	**Ag-ELISA**		**Overall status**	Dead or **euthanized
	**ELISA**	**IFA**	**ELISA**	**IFA**		**liver**	**serum**		
*2182*	-	<80	-	<80	-	+	+	Ag + NT -	euthanized
*2612*	-	<80	-	<80	-	+	+	Ag + NT -	euthanized
*2408*	+	10240	-	<80	-	*ND	+	Ag + NT -	^#^NR
*2615*	+	2560	-	<80	-	+	+	Ag + NT -	euthanized
*2669*	+	2560	-	<80	-	+	+	Ag + NT -	euthanized
*2739*	+	1280	-	<80	-	+	+	Ag + NT -	euthanized
*2921*	+	1280	-	<80	-	+	+	Ag + NT -	euthanized
*2644*	-	80	-	<80	-	+	ND	Ag + NT -	euthanized
*2728*	+	1280	-	<80	-	+	ND	Ag + NT -	euthanized
*2721*	-	<80	-	<80	80	+	+	Ag + NT +	euthanized
*2972*	-	<80	-	<80	160	+	+	Ag + NT +	dead
**2180**	+	1280	+	320	80	-	-	Ag - NT +	dead
**2181**	+	80	+	80	320	-	-	Ag - NT +	dead
**2189**	+	1280	+	320	320	-	-	Ag - NT +	NR
**2190**	+	160	+	640	640	-	-	Ag - NT +	NR
**2191**	+	640	+	320	320	-	-	Ag - NT +	NR
**2195**	+	2560	+	640	320	-	-	Ag - NT +	NR
**2404**	+	1280	+	160	160	-	-	Ag - NT +	dead
**2693**	+	160	+	<80	320	-	-	Ag - NT +	euthanized
**2696**	+	2560	+	160	320	-	-	Ag - NT +	euthanized
**2713**	+	5120	+	320	160	-	-	Ag - NT +	euthanized
2719	-	80	-	<80	-	ND		ND	NR
832	-	<80	-	<80	-	ND		ND	NR
888	-	<80	-	<80	-	ND		ND	NR
1134	-	<80	-	<80	-	ND		ND	NR
2636	-	<80	-	<80	-	ND		ND	euthanized
2194	+	5120	-	80	-	ND		ND	dead
No. of positive samples	17/27	19/27	10/27	10/27	12/27 (44%)	11/21 (52%)			
	19/27 (70%)		11/27 (41%)						

*ND: not determined, **euthanized: monkeys were euthanized regardless of clinical manifestation. ^#^*NR*: not recorded, *Ag* +: antigen positive, *Ag* -: antigen negative, *NT*+: neutralization antibody positive, *NT*-: neutralization antibody negative. The specimens with case IDs written in italics were considered in acute phase. The specimens with case IDs written in bold were considered in convalescent phase.

We next attempted to detect the neutralization (NT) antibody response in the sera of RESTV-infected cynomolgus macaques. The VSV pseudotype RESTV GP_1,2_ (VSV-RESTV-GP_1,2_/GFP) was used for the NT assay [17]. Twelve serum samples (12/27, 44%) neutralized the VSV-RESTV-GP_1,2_/GFP infection, with NT titers ranging from 80 and 640 (#2721, 2972, 2180, 2181, 2189, 2190, 2191, 2195, 2404, 2693, 2696 and 2713) (Table 1). The anti-GP_1,2_ IgG were not detectable in #2721 and #2972 by IgG ELISA, while those samples both had a neutralizing activity. These two specimens showed a positive response for viral antigen in the Ag-capture ELISA and were thus considered to be collected in an early seroconversion phase.

All cynomolgus macaques at the facility were euthanatized regardless of clinical status and there was a possibility that some of the cynomolgus macaques had combined infection with simian hemorrhagic fever virus (SHFV) in the animal facility [18]. Therefore, immune responses against RESTV did not always reflect the clinical manifestation. For these reasons, we defined “convalescent” or “non-convalescent” based only upon serological findings.

It is noteworthy that, among the serum samples that were positive for viral antigen and negative for the NT antibody (Ag +, NT -), all nine serum samples were negative for anti-GP_1,2_ antibodies, whereas only two samples were negative for anti-NP antibodies. On the other hand, all of the ten serum samples that were negative for viral antigen and positive for the NT antibody (Ag -, NT +) were positive for both anti-GP_1,2_ and anti-NP antibodies. This finding indicates that the anti-GP_1,2_ antibody may therefore increase in cynomolgus macaques in the convalescent phase, while anti-GP_1,2_ antibody is rarely detectable in the acute viremic phase of infection.

### Multiplex assay for cytokines and chemokines in the cynomolgus macaque sera

Ebola virus infection triggers the expression of several proinflammatory cytokines/chemokines [11,19,20]. To examine whether the convalescence from the RESTV infection correlates with the circulating proinflammatory cytokines/chemokines, eleven RESTV-infected cynomolgus macaque serum samples were subjected to a multiplex cytokine analysis. Since we do not know when the infection occurred for each cynomolgus macaque, it is still unclear whether the sera represented an early or late stage of infection. We used seven convalescent phase sera (Ag-, NT +: #2404, 2181, 2189, 2693, 2696, 2713, 2180), and four acute viremic phase sera (Ag +, NT -: #2182, 2612, 2739, 2921). Among the 27 serum samples, only these 11 serum samples were available for multiplex assay. Since the sera were heat-inactivated at 56°C for 30 min prior to being subjected to the multiplex analysis, some cytokines, such as GM-CSF and IL-2, which were previously shown to be elevated in some RESTV infected cynomolgus macaques [14], could not be measured in the assay.

**Figure 1:**
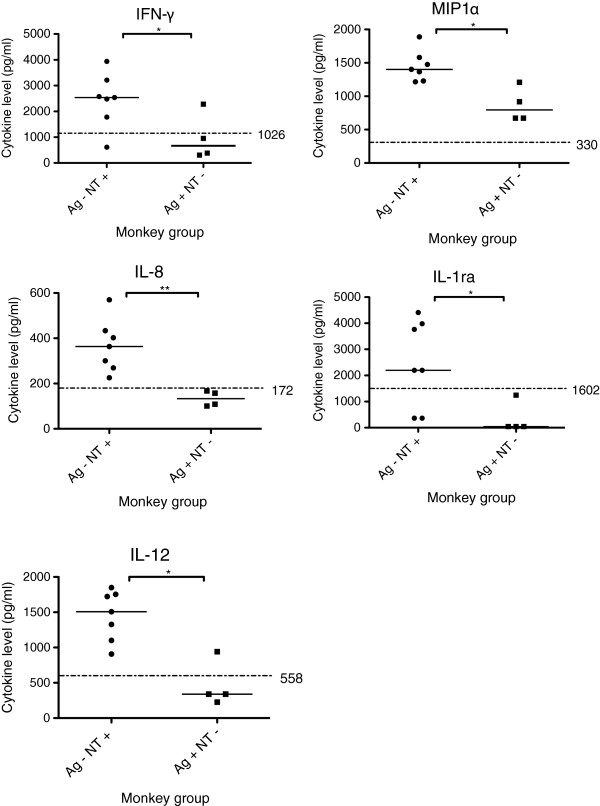
**Profiles of the serum cytokine and chemokine concentrations in Reston virus-infected cynomolgus macaques.** The serum concentrations of IFN-γ, IL-8, IL-12, MIP1α, and IL-1ra. The concentrations of these proinflammatory cytokines/chemokines (IFN-γ, IL-8, IL-12, MIP1α) and the anti-inflammatory cytokine (IL-1ra) were significantly higher in convalescent (Ag - NT +) than in non-convalescent sera (Ag + NT -). Each dot represents one sample, and dashes (-) represent the median values. * indicates p < 0.05, ** indicates p < 0.005 (Mann Whitney test). Broken lines and the numbers written aside indicate the average concentrations of cytokine and chemokine in negative control cynomolgus macaques (n = 13).

**Figure 2:**
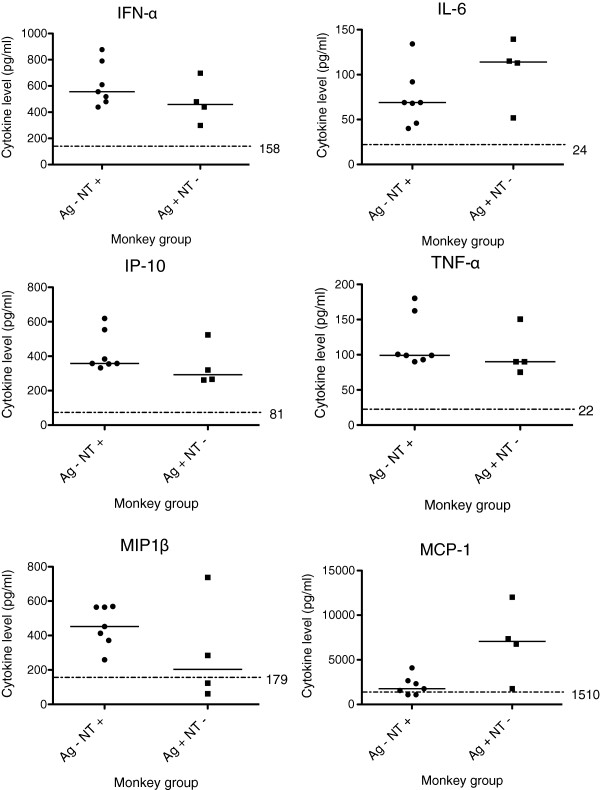
**Profiles of the serum cytokine and chemokine concentrations in Reston virus-infected cynomolgus macaques.** The IFN-α, IP-10, TNF-α, MIP1β, IL-6 and MCP-1 concentrations. The serum concentrations of these cytokines/chemokines (IFN-α, IP-10, TNF-α, MIP1β) did not differ significantly between the convalescent cases (Ag - NT +) and the non-convalescent cases (Ag + NT -). The concentration of the anti-inflammatory cytokine, MCP-1, was lower in convalescent than in non-convalescent cynomolgus macaques (Mann Whitney test). Each dot represents one sample, and dashes (-) represent the median values. Broken lines and the numbers written aside indicate the average concentrations of cytokine and chemokine in negative control cynomolgus macaques (n = 13).

We found that concentrations of several proinflammatory cytokines/chemokines (e.g., IFNγ, IL8, IL-12, IL-1ra, and MIP1α) were significantly higher in convalescent than in acute phase sera (Figure 1). This observation is similar to the previous studies showing elevated concentrations of proinflammatory cytokines/chemokines in the convalescent or asymptomatic human cases [12,13]. In contrast, the concentrations of the five cytokines/chemokines (e.g., IFNα, IP-10, MIP1β, IL-6, and TNFα) did not differ significantly between the two groups (Figure 2). Furthermore, the concentration of MCP-1, one of the proinflammatory chemokines, was lower in the convalescent than in the non-convalescent sera (Figure 2). These data indicated that IFN-γ, IL-8, IL-12, IL-1ra or MIP1α might therefore be involved with the host immune responses in the convalescent phase of RESTV infection.

## Discussion

We previously developed a RESTV NP-specific IgG-ELISA and IFA that proved to be useful for the seroepidemiological studies of cynomolgus macaques during the RESTV epizootic in the Philippines in 1996 [6-8]. The assays based on recombinant NP are sensitive for the detection of RESTV-specific antibodies. On the other hand, anti-GP_1,2_ antibodies are elicited in EBOV-infected human cases and are believed to have protective roles against lethal EBOV infection [21,22]. In the present study, in order to gain insight into the IgG responses during the recovery from infection with RESTV, anti-NP, anti-GP_1,2_, and neutralizing antibodies and the level of viremia in the serum specimens were analyzed. The data presented herein showed that the anti-GP_1,2_ response, rather than the anti-NP response, was correlated with both the lack in viremia and the neutralizing activities in the sera of RESTV-infected cynomolgus macaques. There may be at least two possibilities for the lack of anti-GP_1,2_ IgG in the acute phase samples. It is known that soluble GP (sGP), which does not contain membrane anchor, is secreted during ebolaviruses infection, and it can absorb the anti-GP_1,2_ antibodies [23]. The other possibility is that apoptosis of lymphocytes is induced during RESTV infection and the resulting host immune responses may thus be abrogated. Although the precise mechanism of action is still unknown, it is likely that no IgG responses to RESTV GP_1,2_ are induced in the cynomolgus macaques during the acute phase of infection.

Since the cynomolgus macaques at the facility where the RESTV epizootic occurred were euthanized, sequential serum specimens from each cynomolgus macaque were not available. It is also difficult to determine when each cynomolgus macaque became infected with RESTV. We found that three specimens that have anti-GP antibodies were obtained from dead monkeys (#2180, 2181 and 2194), however, it is difficult to conclude whether RESTV infection caused their death because of the possibility of succumbing to infection by SHFV or some other agents. It is predicted that, among the serum samples examined here, nine were acute phase samples because they were positive in Ag-ELISA or immunohistochemistry [3,15] (Table 1). On the other hand, all but one (serum #2194) of the anti-GP_1,2_-positive serum samples were Ag negative. Therefore, these cynomolgus macaques were considered to be in the convalescent phase of RESTV infection. In this regard, the presence of the anti-GP_1,2_ antibody is thought to be a useful indicator for convalescence in cynomolgus macaques infected with RESTV.

Aberrant proinflammatory cytokines/chemokines are a significant factor implicated in the disease progression of EBOV- and SUDV-infected human cases and experimentally infected cynomolgus macaques [10,11,24]. In addition, a balanced proinflammatory response is believed to be a critical factor for determining the disease outcome [25,26]. We focused on the circulating inflammatory cytokines/chemokines in RESTV-infected cynomolgus macaques and examined their relationship with convalescence. We thus found the concentrations of several proinflammatory cytokines/chemokines, such as IFNγ, IL8, IL-12, and MIP1α, to be significantly higher in convalescent sera than in non-convalescent sera. Gupta et al. (2012) recently demonstrated that convalescent serum samples obtained from BDBV-infected human cases include high concentrations of IL-1α, IL1β, IL6, TNFα, and MCP-1 [13]. Although the exact profiles of proinflammatory cytokines/chemokines shown in our study are different from those reported by Gupta et al., these differences are considered most likely to be due to differences among ebolaviruses (RESTV vs. BDBV), host species (cynomolgus macaques vs. humans), and differences related to the disease phase when the samples were obtained. It is possible that the upregulation of the proinflammatory innate immune responses contributed to the recovery from RESTV infection in cynomolgus macaques.

In rhesus monkeys experimentally infected with a lethal dose of EBOV, anti-inflammatory cytokines, such as IL-13 and IL-1ra, are highly elevated in the acute phase [11]. In human Ebola VHF patients, increased concentrations of IL-10 and IL-1ra have been shown in fatal cases, thus suggesting that the mixed anti-inflammatory response syndrome (MARS) contributes to the pathogenesis of the hemorrhagic fever caused by ebolaviruses. Since all of the cynomolgus macaques involved in the epizootic were euthanized at the affected facility, the actual fate of the cynomolgus macaques was not clear, and some might have survived the infection. Our data obtained using the sera from cynomolgus macaques in the RESTV epizootic showed higher IL-1ra responses in the convalescent phase than in the non-convalescent phase (Figure 1). There were no significant differences in the concentration of IL-10 between the two groups (data not shown). This suggests that, unlike other ebolaviruses infections, RESTV does not induce MARS, which is characterized by an elevated induction of IL1ra in the acute phase.

In conclusion, we have shown that the anti-GP_1,2_ responses, rather than the anti-NP responses, in cynomolgus macaques naturally infected with RESTV were specifically detected in the convalescent stage of RESTV infection. In addition, a high concentration of proinflammatory cytokines/chemokines was observed in the convalescent phase. Therefore, the anti-GP_1,2_ response and the upregulation of the specific proinflammatory response might be useful indicators of convalescence from RESTV infection in cynomolgus macaques.

## Conclusions

In this study, we analyzed the humoral responses in cynomolgus macaque serum samples collected during the 1996 Reston outbreak in the Philippines and demonstrated that the anti-RESTV GP_1,2_ response and the proinflammatory innate response play significant roles in the convalescence from RESTV infection in cynomolgus macaques.

## Methods

### Sera

Twenty-seven cynomolgus macaque serum samples were obtained from the cynomolgus macaque facility in the Philippines where the 1996 RESTV epizootic occurred [27]. The serum specimens in the affected facility were collected under quarantine of the Philippines. Nineteen of the 27 samples were previously subjected to an antigen capture ELISA. Nine of the samples were found to be RESTV antigen-positive, and the remaining 10 were considered to be antigen-negative [15]. The serum specimens were treated at 56°C for 30 minutes and virus in the cynomolgus macaque serum samples were inactivated. As negative controls for the IgG-ELISA and IFA, we used serum samples from 102 cynomolgus macaques collected at the Tsukuba Primate Research Center (TPRC) in Japan. As positive controls for the IgG-ELISA and IFA, two rabbits were immunized four times with the histidine-tagged ectodomain of the RESTV glycoprotein (GP_1,2_) (RESTV GP_1,2_ΔTM). The histidine-tagged RESTV GP_1,2_ΔTM of a 1996 RESTV [28] was prepared and purified as described below. The sera were collected from the rabbits, inactivated, and stored at 4°C until use. The experiments with animals were performed in accordance with the Animal Experimentation Guidelines of the National Institute of Infectious Diseases. The protocol was approved by the Institutional Animal Care and Use Committee of the institute (Permit number: 990163 and 109075).

### Expression and purification of the recombinant RESTV GP_1,2_ ectodomain

A recombinant baculovirus that expresses RESTV GP_1,2_ΔTM was used to prepare recombinant RESTV GP_1,2_ for the IgG-ELISA [16]. Briefly, a recombinant baculovirus carrying the ectodomain of RESTV GP_1,2_ (DDBJ accession no. AB050936) with histidine-tag sequences at its 3’-terminus was infected into Tn5 cells at a multiplicity of infection (moi) of 1. The cells were collected, washed with PBS, and then lysed in PBS containing 1% Nonidet P40 (NP-40) on ice for 15 min. After being centrifuged, the recombinant RESTV GP_1,2_ΔTM was purified with Ni^2+^-agarose beads (QIAGEN, Hilden, Germany) and His Bind Kits (Novagen, Darmstadt, Germany). The purified recombinant RESTV GP_1,2_ΔTM was used for the IgG-ELISA specific for RESTV GP_1,2_. Lysates of Tn5 cells infected with baculovirus with a deleted polyhedrin gene, Ac-ΔP, were similarly processed and then used as negative control antigen in the IgG-ELISA described below.

### RESTV GP_1,2_-specific IgG-ELISA

Ninety-six well plates were coated with the RESTV GP_1,2_ΔTM or with negative control antigen in 100 μl of PBS and incubated overnight at 4°C. The plates were washed three times with PBS containing 0.05% Tween 20 (PBS-T), and then 200 μl of PBS-T containing 5% skim milk (SKIM-PBS-T) was added to each well and incubated for 2 hr at 37°C. The cynomolgus macaque sera were diluted at 1:100, 1:400, 1:1,600, and 1:6,400 in SKIM-PBS-T, and the hyperimmune rabbit sera were four-fold serially diluted from 1:1,000 to 1:64,000 in SKIM-PBS-T. One hundred microliters of each serum dilution was added to the antigen-coated wells and incubated for 1 hr at 37°C. After they were washed three times with PBS-T, the wells were further reacted with either HRP-conjugated goat anti-human IgG (H + L) (Lot:60504974, ZyMED) or HRP-conjugated goat anti-rabbit IgG (H + L) (Lot:398581A, ZyMED) at a dilution of 1:1,000 in SKIM-PBS-T. After being washed three times again with PBS-T, the ABTS substrate (Roche Diagnostics) was added to the wells. Then, the plates were incubated for 30 minutes at 37°C, and the OD values of the wells at 405 nm were measured. Adjusted OD values were calculated by subtracting the OD value of the wells coated with the negative control antigen from that of the wells coated with RESTV GP_1,2_ΔTM.

### RESTV NP-specific IgG-ELISA

The NP-specific IgG-ELISA, which is similar to the GP_1,2_-specific ELISA except for the purified recombinant RESTV NP with a histidine tag at the C-terminus, has been previously reported [6,16].

### ELISA index and determination of the cut-off value for the IgG-ELISA

The sum of the OD values of serum dilutions at 1:100, 1:400, 1:1,600, and 1:6,400 for each specimen was calculated and designated as an “ELISA index” in the IgG-ELISA. The mean plus three standard deviations (SD) of the ELISA indices for the IgG-ELISAs was calculated using serum samples from uninfected TPRC cynomolgus macaques and was used as the cut-off value for the IgG-ELISAs.

### Indirect immunofluorescent antibody assay (IFA) specific for RESTV NP and GP_1,2_

The IFA specific for RESTV NP was reported previously [8]. In the present study, a RESTV GP_1,2_-specific IFA was established using stably RESTV GP_1,2_-expressing HeLa cells. HeLa cell line was purchased from the American Type Culture Collection and used. The RESTV GP_1,2_ cDNA of a 1996 RESTV was subcloned into a mammalian expression plasmid, pKS336, to generate pKS336-RESTV-GP_1,2_. The HeLa cells expressing RESTV GP_1,2_ were selected in a medium containing 2 μg/ml of blasticidin-S-hydrochloride (Sigma, St. Lois, MO) after transfection with pKS336-RESTV-GP_1,2_ using the FuGENE HD Transfection Reagent (Roche Diagnostics, Germany). The cells were trypsinized, washed with PBS, and mixed with normal HeLa cells, and were then spotted on 14 well Teflon-coated glass slides, air dried, and fixed with acetone at room temperature for 5 min. The slides were stored at -80°C until use.

The slides were thawed and dried just before use. The serum specimens were 2-fold serially diluted in PBS, and a 20 μl aliquot of each dilution was applied to the wells of the antigen slides and incubated at 37°C for 1 hr in a humidified chamber. Then the antigen slides were washed with PBS and reacted with 20 μl per well of FITC-conjugated goat anti-human IgG (H + L) (ZyMax lot: 415460A, Invitrogen, CA, U.S.A.) for cynomolgus macaque sera and FITC-conjugated goat anti-rabbit IgG (H + L) (ZyMax lot: 402686A, Invitrogen, CA, U.S.A.) for rabbit hyperimmune sera at a dilution of 1:100. After incubation at 37°C for 1 hr, the slides were washed with PBS and covered with micro cover glasses. The slides were examined for the staining pattern under a fluorescent microscope. The antibody titer in the IFA was determined as the reciprocal of the highest dilution showing positive staining.

### RESTV neutralization (NT) assay using VSV-RESTV-GP_1,2_/GFP

The VSV pseudotype bearing RESTV GP_1,2_, VSV-RESTV-GP_1,2_/GFP was generated essentially according to the method described for the VSV pseudotype bearing SARS-CoV S protein [29], except that pKS336-RESTV-GP_1,2_ was used in the present study [17]. Briefly, 293 T cells were prepared in 24 well plates at 20-30% confluency. The cells were transfected with pKS336-RESTV-GP_1,2_ using FuGENE HD. The cells were then cultured for 24 hr and inoculated with VSV ΔG*/GFP pseudotyped with the VSV-G protein at a moi of 5, adsorbed for 1 hr at 37°C, and then washed with DMEM-5% FCS and cultured for 24 hr. The culture supernatants were collected and centrifuged at 1,000 rpm to remove cell debris. Thereafter, the supernatants were stored at -80°C as VSV-RESTV-GP_1,2_/GFP. The infectivity titer of VSV-RESTV-GP_1,2_/GFP, harboring the VSV ΔG*/GFP genome, was determined by counting the number of GFP-positive cells under a fluorescent microscope upon infection into Vero E6 cells, as described previously. Briefly, VSV-RESTV-GP_1,2_/GFP was 3.2 (0.5 log_10_)-fold serially diluted with DMEM-5% FCS and then inoculated to Vero E6 cells seeded in 96 well culture plates. The cells were incubated at 37°C in a CO_2_ incubator for 24 hr. Then, GFP-positive cells were detected and counted under a fluorescent microscope (BZ-9000; KEYENCE, Osaka, Japan), and the infectious units (IU) of the pseudotyped VSV were calculated.

The serum samples were serially diluted in DMEM-5% FCS, and a 50 μl aliquot of each dilution was mixed with the same volume of DMEM-5% FCS containing 1,000 IU of VSV-RESTV-GP_1,2_/GFP and incubated for 1 hr at 37°C. The mixture was inoculated into Vero E6 cells and incubated for 24 hr. The number of GFP-positive infected cells was counted, and serum dilutions with 50% neutralization (NT_50_) were identified.

### Multiplex assay for cytokines and chemokines in the cynomolgus macaque sera

Eleven RESTV-infected cynomolgus macaque serum samples were inactivated at 56°C for 30 min, diluted 1:10 in the assay diluent supplied with the Human Cytokine 25-Plex antibody bead kit (Invitrogen, CA), and were subjected to a multiplex cytokine analysis using a Luminex 100 instrument (Luminex Co., Austin, TX) according to the manufacturer’s instructions. This Human Cytokine 25-Plex antibody bead kit was previously used to cynomolgus macaque sera and the cross-reactivity was confirmed [30]. As negative controls, we used sera from 13 cynomolgus macaques bred at the TPRC and investigated the cytokine concentrations of these serum samples.

## Abbreviations

Ag: Antigen; BDBV: Bundibugyo ebolavirus; EBOV: Ebola virus; ELISA: Enzyme-linked immunosorbent assay; GP_1,2_: Glycoprotein; GP_1,2_ΔTM: Ectodomain of the RESTV glycoprotein; VHF: Viral hemorrhagic fever; IFA: Immunofluorescent antibody assay; MARS: Mixed anti-inflammatory response syndrome; NP: Nucleoprotein; NP-40: Nonidet P40; NT: Neutralization; PBS-T: PBS containing 0.05% Tween 20; RESTV: Reston virus; SD: Standard deviation; SHFV: Simian hemorrhagic fever virus; SKIM-PBS-T: PBS-T containing 5% skim milk; TPRC: Tsukuba Primate Research Center; VSV: Vesicular stomatitis Indiana virus; VSV-RESTV-GP_1,2_/GFP: VSV pseudotype bearing RESTV GP_1,2_.

## Competing interests

The authors declare that they have no competing interests.

## Authors’ contributions

ST, TI, SF, and SM designed the experiments and analyzed the experimental data. ST and SF prepared the manuscript. SM supervised the experiments and helped draft the manuscript. YS, SW, and II helped to perform the experiments. NN performed the multiplex assay. YY and MM prepared the serum samples. TI, TM, YI, MS, HA and SK supervised the experiments. All authors have read and approved the final manuscript.
